# Effects of Blood Flow Restriction Training in Patients before and after Anterior Cruciate Ligament Reconstruction: A Systematic Review and Meta-Analysis

**DOI:** 10.3390/healthcare12121231

**Published:** 2024-06-20

**Authors:** Eduardo Fraca-Fernández, Luis Ceballos-Laita, Héctor Hernández-Lázaro, Sandra Jiménez-del-Barrio, María Teresa Mingo-Gómez, Ricardo Medrano-de-la-Fuente, Ignacio Hernando-Garijo

**Affiliations:** 1 Faculty of Health Sciences, University of Valladolid, 42004 Soria, Spain; eduardo.fraca@uva.es (E.F.-F.); luis.ceballos@uva.es (L.C.-L.); hector.hernandez.lazaro@uva.es (H.H.-L.); sandra.jimenez.barrio@uva.es (S.J.-d.-B.); mariateresa.mingo@uva.es (M.T.M.-G.); ignacio.hernando@uva.es (I.H.-G.); 2 Clinical Research in Health Sciences Group, University of Valladolid, 42004 Soria, Spain

**Keywords:** BFR, ACL, rehabilitation, muscle strength, knee function

## Abstract

(1) Objective: To examine the effects of blood flow restriction (BFR) training on muscle strength, cross-sectional area and knee-related function in patients selected for anterior cruciate ligament reconstruction (ACLR). (2) Methods: A literature search was conducted in PubMed, PEDro, Cochrane Library, Web of Science, SCOPUS, and ProQuest databases until 20 May 2024. Controlled clinical trials comparing the effects of BFR training with unrestricted training in patients before or after ACLR were selected. The GRADE approach was used to assess the degree of certainty for each meta-analysis. (3) Results: Ten studies were included (n = 287 participants). Standardized mean differences in favor of BFR training applied postoperatively were observed in knee extensor (SMD = 0.79; 95% CI = 0.06 to 1.52; I^2^: 68%) and flexor isokinetic strength (SMD = 0.53; 95% CI = 0.04 to 1.01; I^2^: 0%), and quadriceps cross-sectional area (SMD = 0.76; 95% CI = 0.27 to 1.26; I^2^: 0%). No changes were found in knee extensor isometric strength and knee-related function. The degree of certainty according to the GRADE was very low. (4) Conclusions: Very low degree of certainty suggests that BFR training provides additional benefits to unrestricted training on isokinetic strength and quadriceps cross-sectional area in patients undergoing ACLR.

## 1. Introduction

Anterior cruciate ligament (ACL) tears are one of the most common injuries among recreational and elite athletes [1]. The annual incidence has been estimated at 68.6 ACL tears per 100,000 person-years, affecting mainly young people between the ages of 14 and 25 [2].

ACL reconstruction is the most used option to restore joint stability in ACL tears [2]. The strength deficit and the muscle size loss in the affected leg occur after the ACL tear and are accentuated after surgery, regardless of graft type or concomitant meniscal procedures [3,4,5,6]. A strength loss of 17% preoperatively and close to 40% one month postoperatively has been identified [3,7]. Atrophy of the quadriceps, biceps femoris, and semitendinosus muscles begins after injury and undergoes a fourfold volume loss one month after surgery [3,8]. Similarly, the cross-sectional area of the thigh muscles was also reduced compared to the uninjured leg, both before and after ACL [9]. Some gains occur in the following weeks, but only half of the patients fully recover quadriceps and hamstring muscle strength and size after a year from the reconstruction [3,4].

Muscle strength and size appear to be important contributors to patient recovery. Preoperative and postoperative strength can predict knee-related function 6 months after ACL reconstruction, or upon return to sport [10,11,12]. Furthermore, quadriceps muscle size was positively correlated to ACL volume in a recent study [13]. Thus, treatment should focus on preventing strength loss and atrophy in patients undergoing or scheduled for ACL reconstruction.

The American College of Sports Medicine suggests loads of 70% of the individual’s one-repetition maximum (1RM) to optimize muscle strength and hypertrophy gains, or even higher than 80% 1RM in experienced athletes [14]. Patients with ACL injuries are often unable to tolerate training due to pain intensity, swelling, and impaired knee function, especially near the perioperative period [15]. Moreover, an early return to vigorous training could compromise graft laxity [16], so it is important to develop adequate training procedures for these patients. Blood flow restriction (BFR) therapy is applied to elicit greater muscle activation, and consists of generating partial occlusion through external pressure on a body region. The pressure is often applied with pneumatic cuffs on the proximal limb, while the patient exercises [17,18].

The potential effects of BFR training on muscle fitness have been investigated in different populations with muscle weakness and knee atrophy. BFR training appears to promote larger strength and hypertrophy gains than isolated exercise in patients with knee osteoarthritis, rheumatoid arthritis, and older adults [19,20]. The primary mechanisms underlying the clinical improvements through BFR training are under debate. The hypoxic muscular environment generated by BFR could induce metabolic stress that promotes muscle growth. Furthermore, BFR seems to increase neuromuscular demand during exercise, eliciting the recruitment of fast-twitch muscle fibers [21].

Previous studies have conducted systematic reviews and meta-analyses on the effects of BFR, grouping different musculoskeletal conditions [22,23], but its estimated effect on a clinically homogeneous population remains poorly understood. In recent years, there is emerging evidence proposing BFR training in patients scheduled for ACL reconstruction, given its clinical applicability and its promising effects on muscle strength and hypertrophy. This emerging evidence should be systematically and critically reviewed and reported according to evidence-based guidelines. Therefore, the aim of this systematic review with meta-analysis is to evaluate the effects of BFR training compared to the same unrestricted training on muscle strength, cross-sectional area, and knee-related function in patients before and after ACL reconstruction.

## 2. Materials and Methods

### 2.1. Study Design

This systematic review and meta-analysis followed the Preferred Reporting Items for Systematic Reviews and Meta-Analysis (PRISMA) guideline and Cochrane Recommendations [24]. The study protocol was registered in the International Prospective Register of Systematic Reviews under registry code CRD42022345026.

### 2.2. Search Strategy

The bibliographical search was conducted in PubMed (MEDLINE), the Physiotherapy Evidence Database (PEDro), the Cochrane Library, Web of Science (WOS), and SCOPUS from inception to 20 May 2024. The search strategy was defined using the Population, Intervention, Comparison, Outcomes and Study (PICOs) framework. Search terms were selected based on Medical Subject Headings (MeSH): anterior cruciate ligament, anterior cruciate ligament reconstruction, anterior cruciate ligament injuries, and blood flow restriction therapy. A gray literature search was conducted using Scopus and ProQuest databases, and a hand search was carried out on the reference lists of the included studies. The search strategy for each database is detailed in Appendix A.

### 2.3. Study Selection

The PICOs scheme was employed to frame the research question of whether BFR training applied before or after ACL reconstruction has additional benefits to unrestricted training. The included studies met the following criteria: Population (P), patients scheduled for ACL reconstruction; Intervention (I), BFR techniques combined with exercise therapy; Comparison (C), sham-BFR techniques combined with exercise therapy or isolated exercise therapy; Outcomes (O), isometric strength, isokinetic strength, cross-sectional area, and/or knee-related function; Studies (s), randomized or nonrandomized controlled clinical trials.

Studies were excluded due to the following: (1) intervention comprised therapies other than BFR or exercise; (2) BFR was not applied in combination with exercise therapy; (3) restricted and unrestricted training was different between the comparison groups; (4) primary intervention was pharmacological or surgical treatment; (5) selection criteria were not specified; (6) the design was a conference abstract, dissertation, or book; (7) they were not published in English, Spanish, or French.

After retrieving searches, references were exported to Mendeley Desktop, and duplicates were removed. Two reviewers (E.F. and I.H.) independently assessed the titles and abstracts of each reference to determine potential eligibility. The same reviewers independently evaluated the full texts of potentially eligible studies. Discrepancies between the two reviewers were resolved by a third author (L.C.).

### 2.4. Data Extraction

Both reviewers independently gathered information from each study in a standardized manner adapted from the Cochrane Collaboration. Collected data included (1) participants characteristics; (2) description of interventions administered to experimental and control groups; (3) outcome measures; and (4) results. Qualitative synthesis was used for data analysis, and whenever feasible, quantitative synthesis (meta-analysis) was also performed.

### 2.5. Risk of Bias and Quality Assessment

The PEDro scale was applied to assess the methodological quality of each study. This is an 11-item scale based on the Delphi list developed by Verhagen and Colleagues [25]. The first item (eligibility criteria) focuses on external validity and was excluded from the total score. A score of 7 or higher was categorized as “high” quality and 6 or lower was indicative of “poor quality” [26,27]. The Cochrane Risk of Bias tool was used to assess the risk of bias of the included studies. This tool evaluates potential bias and internal validity across 7 criteria, classifying studies as “low”, “unclear”, or “high” risk [28]. The PEDro scale and the Cochrane Risk of Bias tool have demonstrated reliability in assessing study quality and identifying bias [28,29,30]. The methodological quality of the studies was evaluated by the same independent reviewers, with any discrepancies resolved by a third reviewer.

Two investigators assessed the degree of certainty of each meta-analysis using the Grading of Recommendations Assessment, Development and Evaluation (GRADE) approach. This classification categorizes the evidence as “high”, “moderate”, “low”, or “very low”. The downgrading of the certainty level was based on the presence of risk of bias, inconsistency of results, indirectness of evidence, and imprecision [28,31,32]. Detailed criteria for grading the evidence in each domain are found in Table 1.

### 2.6. Data Synthesis and Analysis

The quantitative synthesis of the results was conducted based on the considered outcomes: isometric and isokinetic strength; cross-sectional area; and knee-related function. When studies used different tools or measurement scales to assess the same outcome, the authors performed inverse variance methods.

Six different meta-analyses were performed for the included outcomes, and subgroup analyses of the studies were performed to compare BFR training versus unrestricted training before surgery or after surgery. Mean and standard deviation (SD) of post-intervention and sample size from each group were extracted. Standard mean differences (SMD) and 95% confidence intervals (95% CI) were estimated based on the post-intervention means and SDs. An SMD between 0.2 and 0.5 indicated a small effect size, between 0.5 and 0.8 a moderate effect size, and >0.8 a large effect size [34].

Data were pooled for meta-analysis using a minimum of two trials deemed clinically homogeneous. Statistical significance was set at *p* < 0.05. The 95% CI of the SMD were displayed in the forest plots. A fixed effects model was considered if there was a common intervention and outcome measures. A random effects model was used when the combination of intervention effects could incorporate an assumption that the studies are not all estimating the same intervention effect [33]. In addition, a leave-one-out analysis was carried out to assess the presence of any potential outlying study that affects the estimate of each meta-analysis. All meta-analyses were performed using RevMan 5.4 software.

## 3. Results

### 3.1. Literature Search and Screening

**Figure 1:**
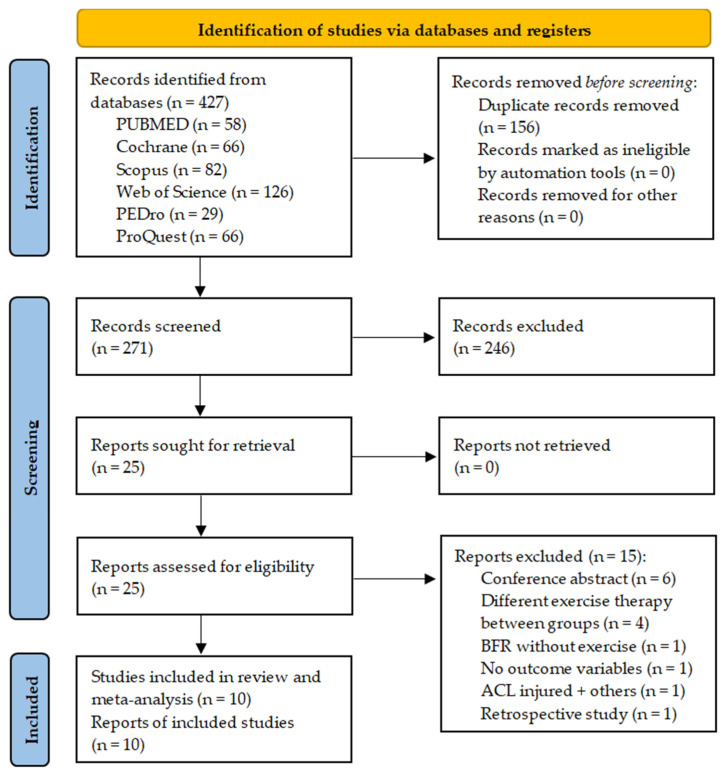
Flow diagram.

Ten studies were selected for inclusion in the qualitative and quantitative synthesis of 427 potentially eligible records retrieved from all databases [35,36,37,38,39,40,41,42,43]. The kappa value of agreement between reviewers was 0.96. The third reviewer resolved discrepancies regarding one study. The selection process is detailed in the PRISMA flowchart diagram (Figure 1).

### 3.2. Characteristics of the Eligible Studies

The included studies comprised 287 participants, the sample sizes ranged from 12 to 45 patients, and the mean ages ranged from 15 to 38 years. Most studies selected patients that underwent ACL reconstruction via semitendinosus–gracilis tendon graft [35,36,38,40,41]. A bone–patellar tendon–bone was predominantly used for ACL reconstruction in three studies [37,39,44]. The type of graft was not specified in two studies [42,43]. Surgical procedures concomitant to ACL reconstruction were only detailed in four studies, all of which involved meniscus surgeries [37,40,41,44]. Sociodemographic and clinical characteristics of the participants are shown in Table 2.

BFR was applied in combination with exercise in all of the studies. Most exercise protocols included low-load weight-bearing exercises involving knee extensions and straight leg raises [35,36,38,39,40,41,42,43,44], with sets ranging from 4 to 6 and repetitions per set ranging between 10 and 40. Only Curran et al. [37] used high-load leg press exercises.

The intervention was performed prior to surgery in four studies [35,39,40,41], post-surgically in five studies [36,37,38,42,43], and perioperatively in one study [44]. The interventions applied before surgery lasted from 1 to 3 weeks and compared BFR with sham BFR [35,40,41] or without BFR [39]. The interventions carried out after surgery lasted for 8 to 14 weeks, except for Iversen et al. [36], which lasted 12 days. All studies compared BFR versus non-BFR, and most interventions started within 2 weeks after surgery [36,38,42,44]. The BFR was applied using a pneumatic cuff applied to the proximal thigh in all studies, and the cuff width was 13.5 or 14 cm. The occlusion pressure ranged from 130 to 180 mmHg in five studies [35,36,38,40,41], four studies used the 80% of the limb occlusion pressure (LOP) [37,39,43,44], Li et al. [43] used the 40% of the LOP, and Jung et al. [42] used the 40% of the systolic blood pressure. Blood flow restriction was intermittently maintained during exercise sets [35,37,40,41,43,44], types [39], or during 5 min intervals [36]. The cuff deflation periods were maintained from 30 to 180 s [35,36,37,39,40,41,43,44]. Sham BFR was applied with the same cuff of the real occlusion inflated at 20 mmHg [35,40,41]. Intervention characteristics of each study are detailed in Table 3.

### 3.3. Outcome Measures

Knee extensor isometric strength was assessed by dynamometry in six studies [37,38,39,40,41,44]. Isokinetic strength of knee extensors and flexors at 60°/s was assessed by dynamometry in five [35,37,38,42,43] and three studies [35,38,42], respectively. Quadriceps cross-sectional area was assessed in four [35,36,38,40], using 1.5T [36,38] or 3T [35,40] MRI systems. Four studies assessed perceived knee function through the International Knee Documentation Committee (IKDC) score [37,42,43,44]. All studies measured the outcomes in the short term (within 4 weeks following the intervention). Outcome measures of each study are shown in Table 2.

### 3.4. Study Quality and Risk of Bias

According to risk of bias Cochrane tool (Appendix B), most of the studies included in this systematic review with meta-analysis described a high risk of selection, performance, and detection bias. According to the PEDro scale (Appendix C), four studies presented high methodological quality [35,36,39,41] and six showed low quality [37,38,40,42,43,44]. All studies provided central and dispersion values, and between-group comparisons for the main outcomes. Only one study provided concealed allocation [37]. Six studies were described as randomized [36,37,38,39,43,44] and four as nonrandomized [35,40,41,42]. Any disagreement between the two reviewers was resolved by the third author.

### 3.5. Synthesis of Results

#### 3.5.1. Effects on Knee Extensor Isometric Strength

**Figure A2:**
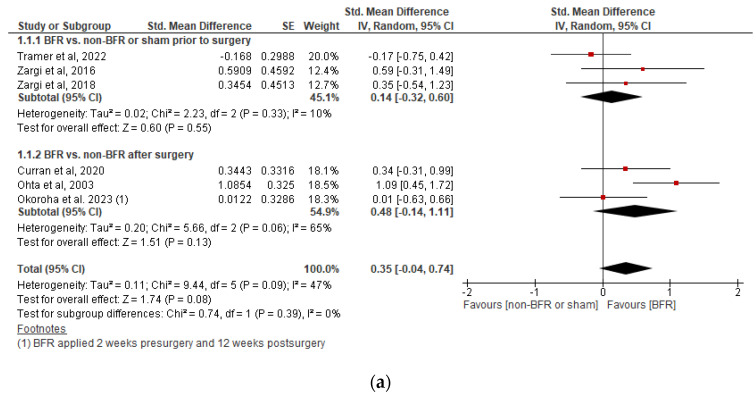

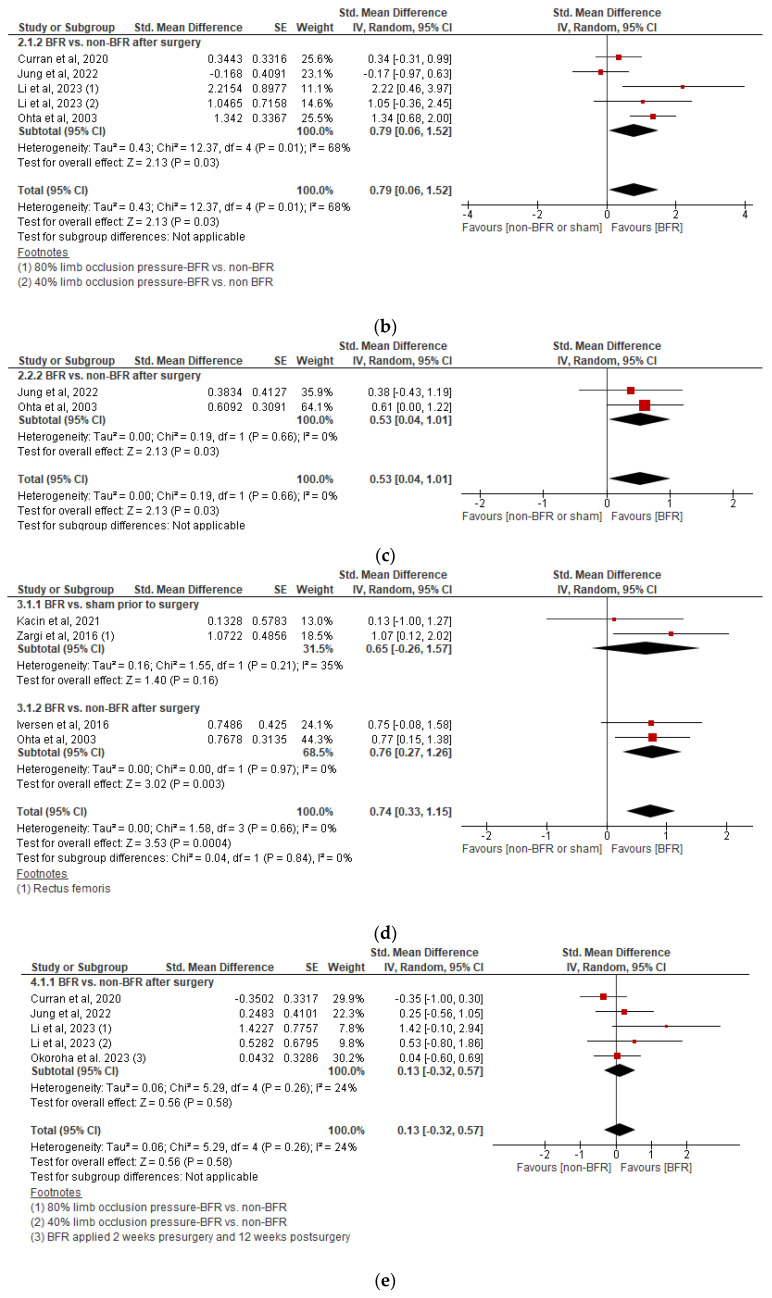
Forest plots. (**a**) Forest plot of knee extensor isometric strength for BFR versus sham or non-BFR intervention. (**b**) Forest plot of knee extensor isokinetic strength for BFR versus sham or non-BFR intervention. (**c**) Forest plot of knee flexor isokinetic strength for BFR versus sham or non-BFR intervention. (**d**) Forest plot of quadriceps cross-sectional area for BFR versus sham or non-BFR intervention. (**e**) Forest plot of perceived function for BFR versus sham or non-BFR intervention [35,36,37,38,39,40,41,42,43,44].

Six studies assessed the effects of BFR compared to unrestricted training on knee extensor isometric strength [37,38,39,40,41,44]. Three studies analyzed the effects of BFR applied preoperatively [39,40,41], and three studies analyzed the effects of BFR applied after surgery [37,38,44]. All of the studies (n = 204) were included in the quantitative synthesis. No statistically significant differences were observed when intervention was applied before ACL reconstruction (SMD = 0.14; 95% CI = −0.32 to 0.60; I^2^: 10%), or when it was applied after surgery (SMD = 0.48; 95% CI = −0.14 to 1.11; I2: 65%). The pooled analysis did not show statistically significant differences (SMD = 0.35; 95% CI = −0.04 to 0.74; I^2^: 47%) (Figure A2a in Appendix D).

#### 3.5.2. Effects on Knee Extensor Isokinetic Strength

Five studies examined the effects of BFR compared to unrestricted training on quadriceps isokinetic strength at 60°/s [35,37,38,42,43]. Four of the studies (n = 128) applied the intervention after surgery and were included in the quantitative synthesis [37,38,42,43]. Statistically significant differences were observed in favor of BFR intervention when BFR was applied after surgery (SMD = 0.79; 95% CI = 0.06 to 1.52; I^2^: 68%) (Figure A2b in Appendix D).

In the leave-one-out analysis, no statistically significant differences were observed (SMD = 0.57; 95% CI = −0.21 to 1.36; I^2^: 56%) when the study by Ohta et al. [38] was considered an outlier.

#### 3.5.3. Effects on Knee Flexor Isokinetic Strength

Knee flexor isokinetic strength at 60°/s was assessed in three studies [35,38,42]. Two studies (n = 68) analyzed the effects of BFR applied after surgery and were included in the quantitative synthesis [38,42]. Statistically significant differences were observed in favor of BFR intervention applied after surgery (SMD = 0.53; 95% CI = 0.04 to 1.01; I^2^: 0%) (Figure A2c in Appendix D).

#### 3.5.4. Effects on Quadriceps Cross-Sectional Area

Two studies assessed the effects of BFR compared to unrestricted training before surgery [35,40], and two studies assessed the effects after surgery [36,38]. All of the studies (n = 100) were included in the quantitative synthesis. No statistically significant differences were shown when BFR interventions were applied before surgery (SMD = 0.65; 95% CI = −0.26 to 1.57; I^2^: 35%), but there were statistically significant differences in favor of BFR applied after surgery (SMD = 0.76; 95% CI = 0.27 to 1.26; I^2^: 0%) and in the pooled analysis (SMD = 0.74; 95% CI = 0.33 to 1.15; I^2^: 0%) (Figure A2d in Appendix D).

#### 3.5.5. Effects on Perceived Knee Function

Four studies measured the effects of BFR compared to unrestricted training after surgery [37,42,43,44]. All of the studies were included in the quantitative synthesis (n = 122), and no statistically significant differences were observed (SMD = 0.13; 95% CI = −0.32 to 0.57; I^2^: 24%) (Figure A2e in Appendix D).

The overall degree of certainty according to GRADE was rated as very low for all the outcomes. The certainty assessment according to GRADE is shown in Table 4.

## 4. Discussion

The aim of this study was to assess the effects of BFR training on strength, cross-sectional area, and knee-related function before and after ACL reconstruction. This systematic review with meta-analysis found very low evidence suggesting that BFR training applied after surgery was more effective than sham BFR training or unrestricted exercise in increasing isokinetic strength at 60°/s and quadriceps cross-sectional area in patients scheduled for ACL reconstruction. There were no statistically significant differences in knee extensor isometric strength and knee-related function.

The results for isokinetic knee extensor strength should be interpreted with caution because when the study by Ohta et al. [38] was treated as an outlier, no statistically significant differences were observed. The sources of bias observed in this study (blinding and allocation concealment) are prevalent across other included studies, and the number of studies in the subgroup meta-analysis is relatively small, as their results could be similarly biased. Nonetheless, clinicians and researchers must remain attentive to the possibility that individual data points may be influencing the stability of the results of the pooled analysis.

The methodological quality of the included controlled trials ranged from 3 to 7 points according to the PEDro scale. Only three studies included in the meta-analyses showed high methodological quality. According to PEDro and Cochrane Risk of Bias tool, common methodological flaws were lack of allocation concealment and blinding of participants and personnel. Four studies were nonrandomized, but three of them were described as quasi-randomized [35,40,41]. In these studies, the authors assigned the first two participants to the BFR group and the next two to the sham group, repeating the process until fulfilled groups. Despite the lack of adequate randomization, these studies reported similar clinical and sociodemographic characteristics between groups at baseline. The overall degree of certainty in the GRADE approach was downgraded mainly due to the low methodological quality of the included studies, heterogeneity among interventions, and small sample sizes. The measurement procedures and the clinical and sociodemographic characteristics of the included participants seem to be sufficiently consistent among studies to conduct a subgroup meta-analysis, but readers should consider the low level of certainty provided by the available evidence.

The pooled meta-analyses revealed moderate effects in favor of BFR training on knee isokinetic strength (extensor: SMD = 0.79; 95% CI = 0.06 to 1.52; flexor: SMD = 0.53; 95% CI = 0.04 to 1.01) and quadriceps cross-sectional area (SMD = 0.74; 95% CI = 0.33 to 1.15). Other meta-analyses also found that BFR could promote benefits in strength and quadriceps cross-sectional area in patients with various knee conditions [22,23]. As main differences compared to these previous meta-analyses, the present study analyzed isometric and isokinetic strength separately, examined the effects of BFR added to exercise therapy, and included exclusively ACL-injured patients. Other systematic reviews suggested that low-load BFR training achieved a greater increase in isokinetic strength at 60°/s than unrestricted low-load training, and similar to high-load training [45,46]. According to this study, BFR training promoted an increase in isokinetic, but not in isometric, knee extensor strength. These results could reflect specific training adaptations, as the training interventions were based on dynamic exercises with concentric and eccentric muscle contractions.

BFR training led to gains in isokinetic strength and cross-sectional area for knee extensors, and strength gains for knee flexors were observed. Two studies analyzed the effects on hamstring cross-sectional area, but these were not included in the meta-analysis due to heterogeneity in the patients’ clinical status and the interventions (before vs. after ACL reconstruction) [35,38]. From a qualitative synthesis, none of these studies demonstrated additional benefits of BFR training in increasing knee flexors cross-sectional area. In this way, changes in muscle size could predict changes in strength by less than 50%, and other factors may be involved, such as musculotendinous stiffness, motor unit recruitment and synchronization, or neuromuscular inhibition [3,47]. The knee-related function, as evaluated by the IKDC, showed no differences between BFR training and unrestricted training. However, for enhanced clinical significance, it would be important if the added advantages in terms of increased strength and cross-sectional area were linked to improvements in perceived function.

Regarding the subgroup analysis, there were also moderate benefits on isokinetic strength and quadriceps cross-sectional area favoring BFR training after ACL reconstruction. Nevertheless, there were no statistically significant differences in any outcome when BFR training was applied before surgery. This may be due to longer interventions when BFR was applied after surgery. Most of these interventions lasted from 8 to 14 weeks [37,38,42,44], whereas they lasted from 1 to 3 weeks when BFR was applied presurgery [35,39,40,41]. Additionally, weakness and atrophy occur after ACL rupture, but are particularly noticeable after surgical repair [3,48]. In this sense, BFR could achieve additional benefits to exercise in highly deconditioned muscles, which is consistent with the first weeks after the reconstruction. Curran et al. [37] was the only study that started BFR intervention late after surgery, and found no benefit through BFR for any outcome. This supports that BFR training does not appear to be more effective than isolated training before or a few months after surgery, when muscle deconditioning is not maximal.

The study samples included young adult individuals with a higher proportion of males in most studies. Regarding age, only Kacin et al. [35] and Curran et al. [37] recruited patients with average ages above 35 or below 25 years old, respectively. Therefore, the sociodemographic characteristics appear consistent across studies, and the clinical differences could depend on the timing of BFR training interventions relative to ACLR. BFR training interventions applied close to the immediate perioperative period used a high number of repetitions (15–40) and low intensity (10–30% 1RM or 40 RM). Curran et al. [37] used a lower number of repetitions (10) and concentric or eccentric phases at high intensity (70% 1RM), probably because patients were able to work at high intensities 10 weeks after surgery. Most studies used three to five sets, and the most commonly used pressure for restriction was 150 mmHg or 80% of limb occlusion pressure. However, there was variability in the way BFR was applied in terms of cuff pressure, inflation, and deflation times; this added to the existing heterogeneity among exercise interventions. The characteristics of the BFR training in patients scheduled for ACL reconstruction are quite similar to in other pathologies, such as knee osteoarthritis, patellofemoral pain and rheumatoid arthritis [19,46].

There were some important limitations. Firstly, the heterogeneity between interventions and measurement time points. Despite performing a subgroup analysis, the pooled analysis of isometric strength and cross-sectional area includes both operated and nonoperated patients who had varying levels of strength, muscle size, and functional status. Furthermore, the BFR and exercise interventions varied among studies. Secondly, the sample sizes were small or insufficient, which may overestimate the results. Thirdly, nonrandomized clinical trials were included due to the small number of available studies, which may have compromised the overall quality of the meta-analyses. Finally, studies comparing low-load BFR training with high-load training were excluded, so relevant results for BFR clinical application may have been omitted.

The main practical applications of the study suggest that BFR may add moderate benefits when applied during exercise in isokinetic strength and quadriceps size in patients undergoing ACL reconstruction. Most interventions applied BFR in the early perioperative period at 80% of LOP or 150 mmHg, ensuring cuff deflation between sets, and combined with low-load training and high repetitions per set (>15).

Future randomized clinical trials with good methodological quality should examine the effects of BFR training in patients with ACL injury. Further research is needed to compare low-load BFR training with high-load training in patients undergoing ACL reconstruction. In addition, future meta-analyses should take into account the baseline muscle fitness and start time of the intervention relative to surgery.

## 5. Conclusions

The results of this systematic review with meta-analysis found very low degree of certainty suggesting that BFR training provides additional benefits compared to the same unrestricted training on isokinetic knee extensor and flexor strength at 60°/s, and quadriceps cross-sectional area in patients selected for ACL reconstruction. These additional benefits likely emerge when BFR is applied after ACL reconstruction rather than before it, but further research is needed. Similar benefits between BFR and unrestricted training were observed in isometric knee extensor strength and knee-related function. These results should be interpreted cautiously due to the heterogeneity and risk of bias among studies.

## Figures and Tables

**Table 1:** Criteria for downgrading the quality of evidence in each domain according to the GRADE approach [33].

Domains of Evidence Quality	Downgrading of Evidence Quality	
	One Level	Two Levels
Risk of bias	>25% of participants from studies with low methodological quality, lack of randomization or allocation concealment, no sample size estimation, or no participants or assessors blinding.	>50% of participants from studies with low methodological quality, lack of randomization or allocation concealment, no sample size estimation, or no participants or assessors blinding.
Inconsistency of results	Significant heterogeneity in outcome measurement or intervention	
	I^2^ value ≥ 50%	I^2^ value ≥ 75%
Indirectness of evidence	Strict selection criteria established to circumvent indirectness evidence domain reassessment.	
Imprecision	95% CI of an SMD > 0.2 points	95% CI of an SMD > 0.5 points
	Sample sizes < 50 individuals	Sample sizes < 30 individuals
	95% CI of the risk ratio crossing the null value	

95% IC: 95% confidence interval; SMD: standardized mean difference.

**Table 2:** Sociodemographic and clinical characteristics of included studies.

Author, Year, Country	Study Design	BFR Group: N Participants, Age ± SD	Comparison Group: N Participants, Age ± SD	N Women/Men	Graft Origin	Concomitant Surgical Procedures	Outcome (Tool)
Curran et al., 2020 USA [37]	RCT	n = 19 17.4 ± 3.5 years	n = 18 15.7 ± 1.3 years	19/15	25 BPTB 6 STG 3 QT	10 MR 3 MX 4 OMI	Knee extensor MVIC and IKS at 60°/s (Biodex 3 dynamometer). Knee-related function (IKDC).
Iversen et al., 2016 Norway [36]	RCT	n = 12 29 ± 7.4 years	n = 12 29.8 ± 8.3 years	10/14	STG	-	Quadriceps CSA (Toshiba Excelart Vantage Atlas 1.5T MRI).
Jung et al., 2022 Korea [42]	Controlled trial	n = 12 30.8 ± 7.6 years	n = 12 27.8 ± 8.4 years	6/18	-	-	Knee extensor and flexor IKS at 60°/s (Biodex 4 dynamometer). Knee-related function (IKDC).
Kacin et al., 2021 Slovenia [35]	Controlled trial	n = 6 38 ± 6 years	n = 6 38 ± 8 years	6/6	STG	-	Knee extensor and flexor IKS at 60°/s (HUMAC NORM dynamometer). Quadriceps and hamstrings CSA (Siemens Magnetom Trio Tim 3T MRI).
Li et al., 2023 China [43]	RCT	G1: n = 8 30.5 ± 5.3 G2: n = 9 29.7 ± 4	n = 6 28.3 ± 5.2	-/-	-	-	Knee extensor IKS to body weight at 60°/s (Biodex 3 dynamometer). Knee-related function (IKDC).
Ohta et al., 2003 Japan [38]	RCT	n = 22 28 ± 9.7 years	n = 22 30 ± 9.7 years	19/25	STG	-	Knee extensor and flexor IKS at 60°/s and IMS (Biodex 3 dynamometer). Quadriceps and hamstrings + adductor CSA (Toshiba Visart 1.5T MRI).
Okohora et al., 2023 USA [44]	RCT	n = 16 25.4 ± 10.6 years	n = 22 27.5 ± 12 years	18/28	30 BPTB 13 STG 3 QT	9 MR 18 MX	Knee extensor IMS (Lafayette hand-held dynamometer). Knee-related function (IKDC).
Tramer et al., 2023 USA [39]	RCT	n = 23 26.5 ± 12 years	n = 22 27 ± 11 years	20/25	34 BPTB 13 STG 3 QT	-	Knee extensor IMS (Lafayette hand-held dynamometer).
Zargi et al., 2016 Slovenia [40]	Controlled trial	n = 10 33 ± 7 years	n = 10 34 ± 10 years	4/16	STG	12 MX	Rectus femoris CSA (Siemens 3T MRI). Knee extensor MVIC (S2P Isometric Knee Dynamometer).
Zargi et al., 2018 Slovenia [41]	Controlled trial	n = 10 34 ± 6 years	n = 10 35 ± 5 years	4/16	STG	12 MX	Knee extensor MVIC (S2P Isometric Knee Dynamometer).

BPTB: bone–patellar tendon–bone; CSA: cross-sectional area; IKDC: International Knee Documentation Committee; IKS: isokinetic strength; IMS: isometric strength; MR: meniscal repair; MRI: magnetic resonance image; MVIC: maximal voluntary isometric contraction; MX: meniscectomy; OMI: other meniscal intervention (abrasion, debridement, or multiple surgery); QT: quadriceps tendon; STG: semitendinosus–gracilis tendon.

**Table 3:** Intervention characteristics of included studies.

Author, Year	Occlusion Tool (Cuff Width)	Occlusion Area	Pressure Level	Comparison Group	Intervention Duration	Common Intervention in Both Groups
Curran et al., 2020 [37]	Delfi Easi-Fit Tourniquet Cuff	Proximal thigh	80% LOP	Non-BFR	8 weeks Start: 10 weeks after surgery	16 sessions, 2/week. Single-leg press. 5 × 10 reps, 2 to 5 sets, 70% 1RM concentric-20% 1RM eccentric, or 20% 1RM concentric-70% 1RM eccentric.
Iversen et al., 2016 [36]	Delfi low pressure cuff (14 cm width)	Proximal thigh	130 to 180 mmHg	Non-BFR	12 days Start: 2 days after surgery	24 sessions, 2/day. Isometric quadriceps contractions, leg mobility, straight leg raise. 5 × 20 reps.
Jung et al., 2022 [42]	Smart Tool Plus single-chamber pneumatic cuff	Proximal thigh	40% systolic blood pressure	Non-BFR	12 weeks Start: 3 days after surgery	36 sessions, 3/week, 60 min/session. ROM, weight bearing, closed and open kinetic chain exercises. 1 × 30 reps, 3 × 15 reps, 10–30% 1RM.
Kacin et al., 2021 [35]	Ischemic Trainer double-chamber cuff (13.5 cm width)	Proximal thigh	150 mmHg	Sham-BFR at 20 mmHg	3 weeks Start: Before surgery	9 sessions, 3/week. Knee flexion and extension of the injured leg. 4 × 40 reps, 40RM.
Li et al., 2023 [43]	AirBands	Proximal thigh	G1: 80% LOP G2: 40% LOP	Non-BFR	8 weeks Start: >8 weeks after surgery	16 sessions, 2/week, 60 min/session. Isometric quadriceps contractions, squats, lunges, walking, cycling. 1 × 30 reps, 3 × 15 reps, 10–20 kg and green or red Therabands for weight-bearing exercises.
Ohta et al., 2003 [38]	Hand-pump tourniquet	Proximal thigh	180 mmHg	Non-BFR	14 weeks Start: 2 weeks after surgery	84 sessions, 6/week. Straight leg raise, hip abduction and adduction, half squat and step-up weight-bearing exercises (6–14 kg), knee-bending and walking exercises. 1–3 × 20 reps.
Okoroha et al., 2023 [44]	Smart Tool Plus single-chamber pneumatic cuff	Proximal thigh	80% LOP	Non-BFR	14 weeks Start: 2 weeks before surgery	10 sessions, 5/week. Knee extension, straight leg raise, long arc quads, quarter squats. 1 × 30 reps, 3 × 15 reps.
Tramer et al., 2023 [39]	Smart Tool Plus single-chamber pneumatic cuff	Proximal thigh	80% LOP	Non-BFR	2 weeks Start: 2 weeks before surgery	10 sessions, 5/week. Knee extension, straight leg raise, long arc quads, quarter squats. 1 × 30 reps, 3 × 15 reps.
Zargi et al., 2016 [40]	VariFit Conture Thigh Cuff (14 cm width)	Proximal thigh	150 mmHg	Sham-BFR at 20 mmHg	9 days Start: 10 days before surgery	5 sessions, 3/week. Unilateral resisted knee extension with SL. 6 × 40, 40RM.
Zargi et al., 2018 [41]	VariFit Conture Thigh Cuff (14 cm width)	Proximal thigh	150 mmHg	Sham-BFR at 20 mmHg	1 week Start: 8 days before surgery	5 sessions, 3/week. Unilateral resisted knee extension with SL. 6 × 40, 40RM.

BFR: blood flow restriction; LOP: limb occlusion pressure; RM: repetition maximum; SL: surgical limb.

**Table 4:** GRADE assessment of blood flow restriction compared to control.

Certainty Assessment							N of Patients		Effect		Certainty	Importance
N of Studies	Study Design	Risk of Bias	Inconsistency	Indirectness	Imprecision	Other Considerations	[Blood Flow Restriction]	[Control]	Relative (95% CI)	Absolute (95% CI)		
Knee extensor isometric strength (Nm)												
6	Controlled trials	Very serious ^a,b,c^	Serious ^e^	Not serious	Very serious ^h,j,k^	None	100	104	-	SMD 0.35 SD higher (0.04 lower to 0.74 higher)	⨁◯◯◯ Very low	CRITICAL
Knee extensor isokinetic strength (Nm at 60°/s)												
4	Controlled trials	Very serious ^a,b,c^	Serious ^e,f^	Not serious	Very serious ^i,l^	None	70	58	-	SMD 0.79 SD higher (0.06 higher to 1.52 higher)	⨁◯◯◯ Very low	CRITICAL
Knee flexor isokinetic strength (Nm at 60°/s)												
2	Controlled trials	Very serious ^a,b,c,d^	Serious ^e^	Not serious	Very serious ^h,l^	None	34	34	-	SMD 0.53 SD higher (0.04 higher to 1.01 higher)	⨁◯◯◯ Very low	CRITICAL
Quadriceps cross-sectional area (cm^2^)												
4	Controlled trials	Very serious ^a,b,c,d^	Not serious	Not serious	Very serious ^h,l^	None	50	50	-	SMD 0.74 SD higher (0.33 higher to 1.15 higher)	⨁◯◯◯ Very low	IMPORTANT
Perceived knee function (IKDC)												
4	Controlled trials	Very serious ^a,c^	Not serious	Not serious	Very serious ^h,j,k^	None	64	58	-	SMD 0.13 SD higher (0.32 lower to 0.57 higher)	⨁◯◯◯ Very low	CRITICAL

CI: confidence interval; SMD: standardized mean difference; a: more than 50% of participants were from studies without blinding of participants or assessors; b: more than 50% of participants were from studies with lack of allocation concealment; c: more than 50% of participants were from studies with low methodological quality; d: more than 50% of participants were from studies without sample size estimation; e: heterogeneity among interventions; f: heterogeneity among results of studies (I^2^ ≥ 50%); h: 95% CI of SMD > 0.2; i: 95% CI of SMD > 0.5; j: 95% CI of the risk ratio crosses the null value; k: sample sizes < 50; l: sample sizes < 30.

**Figure A1:**
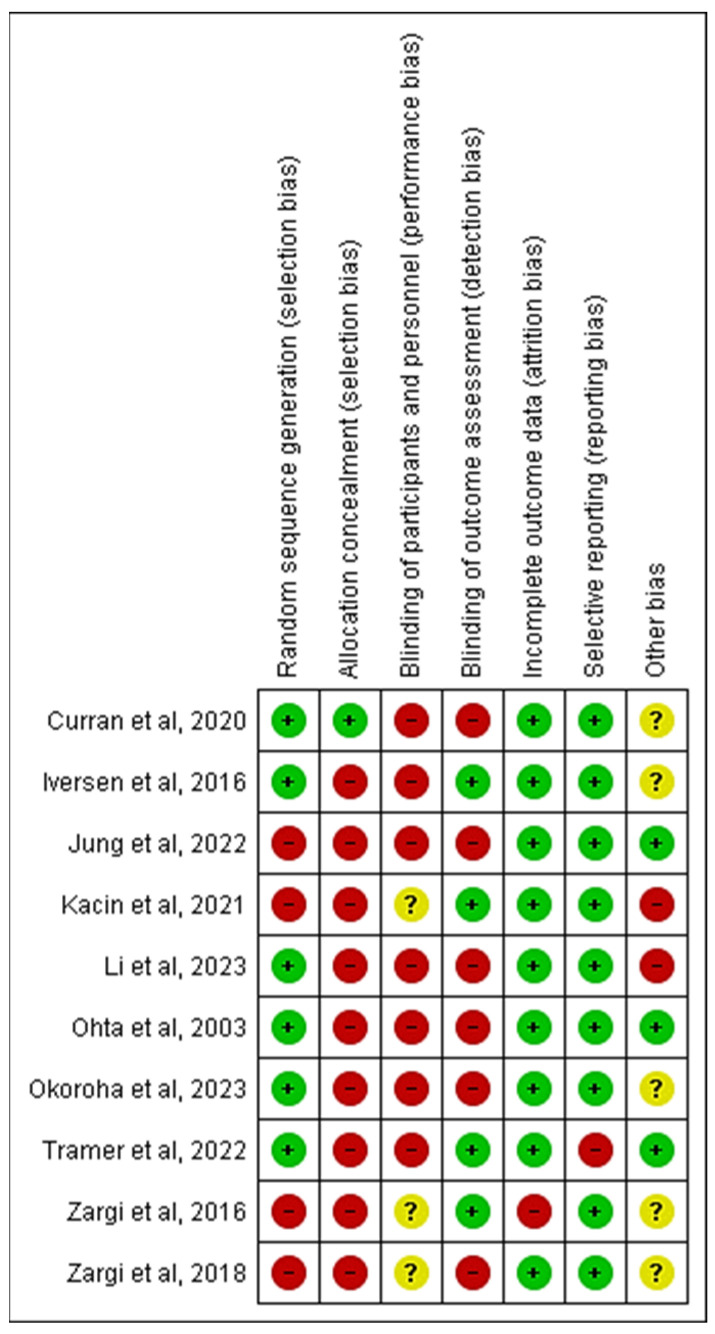

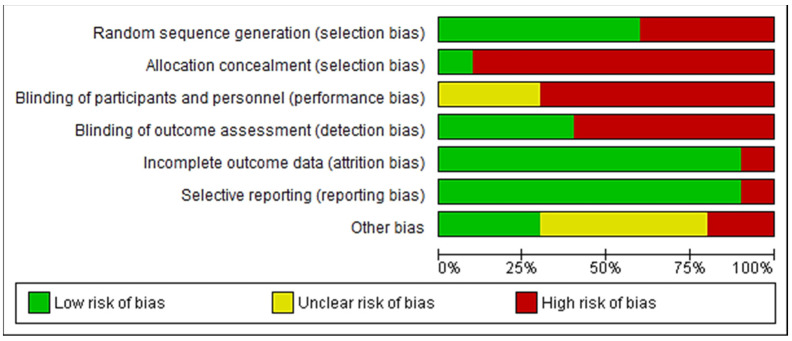
Risk of bias Cochrane tool [35,36,37,38,39,40,41,42,43,44], +: low risk; -: high risk; ?: some concerns.

## Data Availability

Data are contained within the article.

## Appendix A

**Table A1:** Search Strategy.

PubMed		
Search	Strategy	Results
#7	#3 AND #6	58
#6	#4 OR #5	18,374
#5	(“blood”[All Fields] AND “flow”[All Fields] AND “restriction”[All Fields]) OR “blood flow restriction”[All Fields] OR “kaatsu”[All Fields] OR “vascular occlusion”[All Fields] OR ((“occlusion”[All Fields] OR “occlused”[All Fields] OR “occlusions”[All Fields] OR “occlusive”[All Fields] OR “occlusives”[All Fields]) AND (“training”[All Fields] OR “train”[All Fields] OR “trained”[All Fields] OR “trainings”[All Fields] OR “trains”[All Fields])) OR ((“blood vessels”[MeSH Terms] (“blood”[All Fields] AND “vessels”[All Fields]) OR “blood vessels”[All Fields] OR (“blood”[All Fields] AND “vessel”[All Fields]) OR “blood vessel”[All Fields]) AND (“restrict”[All Fields] OR “restricted”[All Fields] OR “restricting”[All Fields] OR “restriction”[All Fields] OR “restrictions”[All Fields] OR “restrictive”[All Fields] OR “restrictiveness”[All Fields] OR “restricts”[All Fields]) AND (“therapeutics”[MeSH Terms] OR “therapeutics”[All Fields] OR “therapies”[All Fields] OR “therapy”[All Fields] OR “therapys”[All Fields]))	18,278
#4	“blood flow restriction therapy”[MeSH Terms]	70
#3	#1 OR #2	31,109
#2	(“anterior”[All Fields] AND “cruciate”[All Fields] AND “ligament”[All Fields] AND “reconstruction”[All Fields]) OR “anterior cruciate ligament reconstruction”[All Fields] OR (“anterior”[All Fields] AND “cruciate”[All Fields] AND “ligament”[All Fields] AND “injuries”[All Fields]) OR “anterior cruciate ligament injuries”[All Fields] OR (“anterior”[All Fields] AND “cruciate”[All Fields] AND “ligament”[All Fields]) OR “anterior cruciate ligament”[All Fields] OR (“acl”[All Fields] AND “injury”[All Fields]) OR “acl injury”[All Fields] OR (“acl”[All Fields] AND “tear”[All Fields]) OR “acl tear”[All Fields] OR (“acl”[All Fields] AND “rupture”[All Fields]) OR “acl rupture”[All Fields]	31,069
#1	“anterior cruciate ligament reconstruction”[MeSH Terms] OR “anterior cruciate ligament injuries”[MeSH Terms] OR “anterior cruciate ligament”[MeSH Terms]	20,659
**Web of Science**		
**Search**	**Strategy**	**Results**
#3	#1 AND #2	126
#2	(blood flow restriction OR kaatsu OR vascular occlusion OR occlusive training OR occlusion training OR blood vessel restriction) (Topic)	158,145
#1	(anterior cruciate ligament OR acl injury OR acl tear OR acl rupture OR acl reconstruction OR acl rehabilitation) (Topic)	44,178
**Scopus**		
**Search**	**Strategy**	**Results**
#3	#1 AND #2	82
#2	TITLE-ABS-KEY (“blood flow restriction” OR “occlusion training” OR “occlusive training” OR “kaatsu” OR “blood vessel restriction” OR “vascular occlusion”)	12,113
#1	TITLE-ABS-KEY (“anterior cruciate ligament” OR “acl injury” OR “acl tear” OR “acl rupture” OR “acl reconstruction” OR “acl rehabilitation”)	39,649
**Cochrane library**		
**Search**	**Strategy**	**Results**
#3	#1 AND 2	66
#2	(blood flow restriction therapy OR blood flow restriction exercise OR blood flow restriction training OR blood flow restriction OR kaatsu OR occlusive training OR blood vessel restriction):ti,ab,kw	2893
#1	(anterior cruciate ligament OR acl):ti,ab,kw	4312
**PEDro**		
**Search**	**Strategy**	**Results**
#5	#2 AND #3	18
#4	#1 AND #3	11
#3	“blood flow restriction”	133
#2	“anterior cruciate ligament”	410
#1	“ACL”	312
	**ProQuest**	**#5**
**Search**	**Strategy**	**Results**
#3	#1 OR #2	66
#2	abstract((“anterior cruciate ligament reconstruction” OR “anterior cruciate ligament injuries” OR “anterior cruciate ligament” OR “acl”) AND (“blood flow restriction” OR “kaatsu” OR “vascular occlusion” OR “occlusive training” OR “occlusion training” OR “ blood vessel restriction”))	42
#1	title((“anterior cruciate ligament reconstruction” OR “anterior cruciate ligament injuries” OR “anterior cruciate ligament” OR “acl”) AND (“blood flow restriction” OR “kaatsu” OR “vascular occlusion” OR “occlusive training” OR “occlusion training” OR “ blood vessel restriction”))	51

## Appendix C

**Table A2:** PEDro scale scores.

Reference	Items											Total Score
	1	2	3	4	5	6	7	8	9	10	11	
Curran et al., 2020 [37]	Y	Y	Y	Y	N	N	N	Y	N	Y	Y	6/10
Iversen et al., 2016 [36]	Y	Y	N	Y	N	N	Y	Y	Y	Y	Y	7/10
Jung et al., 2022 [42]	Y	N	N	Y	N	N	N	Y	N	Y	Y	4/10
Kacin et al., 2021 [35]	Y	N	N	Y	Y	N	Y	Y	Y	Y	Y	7/10
Li et al., 2023 [43]	Y	Y	N	Y	N	N	N	N	N	Y	Y	4/10
Ohta et al., 2003 [38]	Y	Y	N	Y	N	N	N	Y	N	Y	Y	5/10
Okoroha et al., 2023 [44]	Y	Y	N	N	N	N	N	N	N	Y	Y	3/10
Tramer et al., 2023 [39]	Y	Y	N	Y	N	N	Y	Y	Y	Y	Y	7/10
Zargi et al., 2016 [40]	Y	N	N	Y	Y	Y	Y	N	N	Y	Y	6/10
Zargi et al., 2018 [41]	Y	N	N	Y	Y	Y	N	Y	Y	Y	Y	7/10

Out of ten; Y = criterion satisfied and N = criterion not satisfied. 1. Specified eligibility criteria. Not calculated in overall score. 2. Participants randomly allocated to groups. 3. Concealed allocation. 4. Similar groups at baseline. 5. Blinding of participants. 6. Blinding of therapists. 7. Blinding of assessors. 8. Measures of key outcomes obtained from more than 85% of those initially allocated to groups. 9. All participants received the treatment or control condition as allocated or intent-to-treat analysis was performed. 10. Statistical comparisons reported for at least one key outcome. 11. Central and variability measures reported.
